# A Radical Reassessment of the Body in Social Cognition

**DOI:** 10.3389/fpsyg.2020.00987

**Published:** 2020-06-05

**Authors:** Jessica Lindblom

**Affiliations:** Interaction Lab, School of Informatics, University of Skövde, Skövde, Sweden

**Keywords:** radical embodied cognition, social interaction, embodied social cognition, meaning-making, sense-making, situatedness

## Abstract

The main issue addressed in this paper is to provide a reassessment of the role and relevance of the body in social cognition from a radical embodied cognitive science perspective. Initially, I provide a historical introduction of the traditional account of the body in cognitive science, which I here call the cognitivist view. I then present several lines of criticism raised against the cognitivist view advanced by more embodied, enacted and situated approaches in cognitive science, and related disciplines. Next, I analyze several approaches under the umbrella of embodied social cognition. My line of argument is that some of these approaches, although pointing toward the right direction of conceiving that the social mind is not merely contained inside the head, still fail to fully acknowledge the radically embodied social mind. I argue that the failure of these accounts of embodied social cognition could be associated with so-called ‘simple embodiment.’ The third part of this paper focuses on elaborating an alternative characterization of the radically embodied social mind that also tries to reduce the remaining problems with ‘simple embodiment.’ I draw upon two turns in radically embodied cognitive science, the enactive turn, and the intersubjective turn. On the one hand, there is the risk of focusing too much on the individual level in social cognition that may result in new kinds of methodological individualism that partly neglect the social dimension. On the other hand, socially distributed and socially extended approaches that pay more attention to the dynamics within social interaction may encounter the risk of ignoring the individual during social interaction dynamics and simultaneously not emphasizing the role of embodiment. The approach taken is to consider several ways of describing and incorporating the (individual) social mind at the social level that includes language. I outline some ideas and motivations for how to study and expand the field of radical embodied social cognition in the future, as well as pose the ubiquitous hazard of falling back into a cognitivism view in several ways.

## Introduction

Social cognition is an established research field that encompasses several theoretical approaches to describe and study how the social mind works. Hence, there is an intense and ongoing questioning about the role and relevance of the body in social interaction and cognition within cognitive science and related disciplines, and currently there is no single, simple answer to this question (Lindblom, 2007, 2015a). The mainstream study of cognition has since the inception of cognitive science in the mid-1950s mainly focused on studying individual’s internal mental representations in form of symbol manipulation inside the head (e.g., Fodor, 1975, 1983; Newell and Simon, 1976; Gardner, 1987; Fodor and Pylyshyn, 1988). In that view, cognition is viewed as information-processing of these more or less explicit internal symbolic representations, being the “internal content” of the external world, and almost nothing outside “the skull” is taken into account. This is the still common and dominant view in the study of social cognition, suggesting that humans relate to each other in much the same way as they relate to other parts of the external world, i.e., by having more or less explicit internal (symbolic) representations of each other, which then are manipulated internally (e.g., Kunda, 1999; Quinn et al., 2003; Frith and Wolpert, 2004; Singer et al., 2004; Fiske and Taylor, 2013; Augoustinos et al., 2014). Accordingly, the body is only serving as some kind of input and output device, i.e., a physical interface between internal programs (cognitive processes) and the external world in this centralized view of cognition, where social cognition is considered to take place inside the skull. Thus, cognitive psychology in the form of ‘the computer metaphor for mind’ became equivalent to human cognition. Neisser (1967), among others, stresses that the actual task for cognitive psychologists was to understand the ‘program,’ and not the ‘hardware.’ Gardner (1987, p. 6) characterizes the core of cognitive science in its inception as follows: “*First of all, there is the belief that, in talking about human cognitive activities, it is necessary to speak about mental representations and to posit a level of analysis wholly separate from the biological or neurological, on the one hand, and the sociological or cultural, on the other.*” Altogether, this view falls into the category which here is referred to cognitivism.

### Some Reasons for the Neglect of the Body, Criticism of Cognitivism, and the Re-turn to the Body

Historically, there are several reasons for the widespread neglect of the *body* in mainstream cognitive and social sciences (Lindblom, 2007, 2015a,b). On the one hand, it is a consequence of the Platonic-Cartesian heritage, which has resulted in the view of the mind being located in the brain as the internal locus of rationality, thought, language and knowledge (e.g., Fodor, 1975, 1983; Newell and Simon, 1976; Gardner, 1987; Fodor and Pylyshyn, 1988). Moreover, the opposite dimensions have been mapped on each other, resulting in the dualisms of, for instance, mind/body, mental/behavior, reason/emotion, and the subjective/objective. On the other hand, researchers commonly overlook the role of the body because they are afraid of slipping into *biological reductionism*, and therefore they generally prefer to view the mind as superior to and independent of the body (e.g., Segerstråle, 2003). The dichotomy between mind and body has in turn produced a disjunction between verbal and so-called non-verbal aspects of social cognition. and consequently embodied actions such as body posture, gaze and gesture are still commonly considered to be nothing but the visible outcomes of mental intentions and contents which are transmitted from one mind to another (e.g., Mehrabian, 1972; Burgoon et al., 2016). Furthermore, Trevarthen (1977) points out that a practical motive is another reason for the neglect of dynamical aspects, because bodily movements were difficult to observe properly with the technology of the time, and therefore cognitive science consequently became more of a static science of perception, cognition and action than a science of dynamic interactions. At the same time, cognitivism implies that context, history and culture are “murky concepts” (Gardner, 1987, p. 41) that would only cause problems in the effort to find the ‘essence’ of individual cognition. Instead, it was argued, these aspects could be addressed and integrated when cognitive science had achieved an understanding of the central inner mechanisms of individual cognition (Gardner, 1987; Lindblom, 2007, 2015a).

Starting in the late 1970s, several lines of criticism have arouse about the fundamental assumptions with cognitivism in the study of cognition. One addressed criticism is the need to extend cognitivism by taking into account the neurological aspects of cognition more seriously than before, which is aligned with the argument of the *biological implausibility* of the computer metaphor of mind (Maturana and Varela, 1987; Varela et al., 1991; Dreyfus, 1992; Johnson, 2007; Pfeifer et al., 2014; Ziemke, 2016). A second addressed line of criticism is the lack of connections between the external world and the internal representations that threatens its validity (Searle, 1980; Dreyfus, 1992; Lindblom, 2007, 2015a; Hutto and Myin, 2017). A critical aspect lies in the fact that it is unclear how changes in the brain’s states are in structural correlation with the external world and become about it, i.e., having representational content, the so-called ‘symbol grounding’ problem in artificial intelligence (AI) (Harnad, 1990). Either this happens *via* the existence of an additional homuncular system that decodes between the “inner” and “outer” worlds, or “content” is the complex property that can be transferred between them. This also relates to the problem with the origin and content of mental representations exemplified in AI (e.g., Searle, 1980; Dreyfus, 1992; Ziemke, 1999, 2001; Hutto and Myin, 2017). Searle’s (1980) debated whether the Chinese Room Argument addresses the lack of real understanding in the computer program itself, distinguishing between ‘strong AI’ and ‘weak AI.’ A third line of criticism is the lack of *situatedness* in these explanations, and instead it was argued that cognitive science must go beyond the formal representations and take the body and the surrounding world into account “*since intelligence must be situated it cannot be separated from the rest of human life*” (Dreyfus, 1992, p. 62). This ‘rest of human life’ refers to the body’s influence on cognition, cultural factors, and common sense knowledge, which may be impossible to define explicitly. This aspect become rather obvious in traditional AI or so-called good old fashioned AI (GOFAI) were the cognitivism approach of programming a predefined world resulted in poorly behaving robots while acting in the fuzzy real world, although some computer programs, not robots, could master well-defined and specialized tasks like playing chess (Dreyfus, 1992; Clark, 1997; Pfeifer and Scheier, 1999; Ziemke, 1999, 2001; Lindblom, 2007, 2015a). Mental representations may not be necessary, since it appears probable that humans can, for instance, learn to swim, walk or catch a baseball by developing the necessary movements through practice, without any need to represent the bodily (and muscular) movements in the symbolic structure (Dreyfus, 1992; Thelen and Smith, 1994; Lindblom, 2007, 2015a; Wilson and Golonka, 2013). Moreover, Dreyfus (1992) points out that studies in developmental psychology have demonstrated that learning of specific details takes place on a background of shared sociocultural practices which seem to be picked up in everyday interactions not as facts and beliefs but as acquired socially and bodily skills for being-in-the-world, and these sensorimotor coordinations appear to underlie all the “higher” cognitive functions (Smith et al., 1999; Thelen, 2000; Thelen et al., 2001; Wilson and Golonka, 2013). A fourth line of criticism is raised from the ‘turn to the wild’ approach in cognitive science and human–computer interaction (HCI) (Suchman, 1987; Hutchins, 1995; Hollan et al., 2000; Rogers, 2012; Rooksby, 2013). Suchman (1987) stresses the impact of the momentary circumstances in a situation more than the importance of internal representations of plans. She introduces a new analytic model to study cognition where the relevant *actions* are driven by its context, reframing the issue of *interaction per se* in terms of sense-making practices (Rooksby, 2013). Hutchins (1995) emphasizes that there are unnoticed costs involved when we disregard culture, context and history in human cognition. Instead, we should be viewing it as a socio-cultural process and broaden the unit of analysis to a systems perspective. He argues that “cognitive science made a fundamental category error when it mistook the properties of a person in interaction with a social and material world for the cognitive properties of whatever is inside the person” (Hutchins, 2006, p. 1). Hence, the common theme in the criticisms raised above is that cognitivism, when studying cognition, seldom does a task analysis nor does it take into account what kinds of perceptual, embodied, situational, social and cultural resources and scaffolds are present to solve the actual task (Wilson and Golonka, 2013).

This has resulted in a turn, or rather a re-turn, to embodied and situated alternative views, which have been proposed by several scholars throughout the years. They argue, along similar lines, that cognitivism misinterprets the interrelated connections between brain, body and world in cognition and fails to realize the very nature of cognition (e.g., Maturana and Varela, 1980, 1987; Suchman, 1987, 1993; Varela et al., 1991; Dreyfus, 1992; Hutchins, 1995; Clark, 1997; Smith et al., 1999; Thelen, 2000; Thelen et al., 2001; Gallagher, 2005, 2015, 2017; Johnson, 2007; Lindblom, 2007, 2015a,b; Chemero, 2009, 2013; Wilson and Golonka, 2013; Hutto and Myin, 2017; Fuchs, 2018; Newen et al., 2018). These scholars, among others, emphasize the ways cognition is shaped by the embodied human’s interactions with the surrounding material, social, and cultural world. Some of the most prominent advocators of the embodied cognition and *enactive* approach, Varela et al. (1991, p. 172–173, original emphases) explain the phrase *embodied action* as follows: “By using the term *embodied* we mean to highlight two points: first, that cognition depends upon the kinds of experiences that come from having a body with various sensorimotor capacities, and second, that these individual sensorimotor capacities themselves are embedded in a more surrounding biological, psychological and cultural context. By using the term action we mean to emphasize once again, that sensory and motor processes, perception and action, are fundamentally inseparable in lived cognition. Indeed, the two are not merely linked in individuals, they have also evolved together. In a nutshell, the enactive Varelian approach consists of two points: (1) perception consists in perceptually guided action and (2) cognitive structures emerge from the recurrent sensorimotor patterns that enable action to be perceptually guided.” Varela et al. (1991) are strongly influenced by phenomenology, pragmatism as well as Buddhism. In other words, cognition is for *action* and *action-readiness* (Engel et al., 2013), and the subjective tactile-kinesthetic experience of one’s own moving lived body is the bedrock of thinking (Sheets-Johnstone, 1999). This means that the self-experienced bodily understanding is the elemental and unsurpassable unity of embodied actions. Damasio (1995) points out that the brain and body form an indissociable organism. He claims that the separation between the mind and the brain is only mythical, the separation between them is most likely fictional. In recent years, the cognitive science field has introduced more elaborate views on cognition that Marsh (2006) refers to as DEEDS (Dynamical, Embodied, Extended, Distributed, and Situated) theories of cognition. In a similar vein, Barrett (2015) as well as Newen et al. (2018) refer to 4E-cognition (Embodied, Embedded, Enactive, and Extended), arguing that although they differ from each other in a number of significant ways, the DEEDS and 4E-approaches share and have in common the central idea that cognitive processes emerge from the unique manner in which an agent’s (either human, animal or robot) morphological structure and its sensory and motor capacities enable it to engage successfully with its social and material environment in order to bring fourth adaptive and flexible actions. Hence, the two underlying assumption for the DEEDS and 4E approaches of cognition are: (1) the agent’s embodied interactions matters for intelligence, and (2) the need for broadening the focus and scope of the agent’s cognitive system “beyond the brain.”

However, over the last decades, the main focus in most DEEDS and 4E approaches of cognition has until recently been on the relation between the individual body and its cognitive processes, in interaction with the physical environment, and there is a need to consider the fundamental situated and embodied nature of social interaction and cognition (e.g., Johnson, 2007; Lindblom, 2007, 2015a,b; De Jaegher et al., 2010; Fuchs, 2018; Newen et al., 2018). Lately, an increased interest has been further explored to the social dimension of embodied cognition that ranges from the role of *social embodiment effects* (Barsalou et al., 2003; Niedenthal et al., 2005), *mirror neuron systems* and *embodied simulations* that provide embodied explanations of the traditional concepts of “mindreading” and “theory of mind” (Gallese, 2004; Gallagher, 2005), *speech and gesture as an intertwined symbiotic system* (Lindblom, 2007, 2015a,b) to *participatory* aspects of social understanding and *sense-making practices* (De Jaegher and Di Paolo, 2007; De Jaegher et al., 2010; Kyselo, 2014) to *language* (Cuffari et al., 2015; Di Paolo et al., 2018).

Taking a socially embodied view, the above embodied approaches to social cognition, look rather similar at first glance, but taking a closer look, there are fundamental differences. A central aspect is that although many of these embodied approaches are leaning toward the direction of conceiving the social mind as truly embodied, in many regards they still remain aligned, to various extents, to cognitivism. Clark (1999) distinguishes between *simple embodiment* and *radical embodiment.* In simple embodiment, the traditional foundation of cognitivism is preserved, and the nature of embodiment is merely considered a constraint of the ‘inner’ organization and processing. Radical embodiment goes much further and treats the facts of embodiment as a fundamental shift in the explanation of cognition that is “*profoundly altering the subject matter and theoretical framework of cognitive science”* (Clark, 1999, p. 348). A similar line of argument is addressed by Gallagher (2015), who emphasizes that the ongoing discussion of what really constitutes embodied cognition is needed and may define important differences between embodied cognition theorists, but still holds the stance that the body plays a significant role in cognition. He points out that some scholars are making a more reactionary move, formulating a kind of disembodied version of embodied actions that leaves the body out of it, only focusing on what happens inside the brain. Gallagher denotes these efforts of putting the “body in the brain” as *body snatching.* He urges other proponents of embodied cognition to resist the invasion of body snatchers, i.e., challenging those who neglect the role of the radically enacted body in agent-environment interaction as the fundamental basis *per se* in cognition, a quest that I try to achieve in the rest of this paper, with a particular focus on the social mind.

Chemero (2009, 2013) points out that there are at least two different scientific traditions, which both are commonly referred to as ‘embodied cognitive science’. Chemero denotes one of these traditions as radical embodied cognitive science, which has roots in American naturalism (e.g., the work of Williams James and John Dewey) and Gibson’s ecological psychology, being anti-representationalist and anti-computationalist traditions of eliminativism and pragmatism. Chemero (2009) defines radical embodied cognitive science as “*the scientific study of perception, cognition, and action as necessarily embodied phenomenon, using explanatory tools that do not posit mental representations. It is cognitive science without mental gymnastics*” (p. 29). The other direction is the more mainstream version of embodied cognitive science, i.e., simple embodiment, which is derived from traditional theoretical frameworks that are referred to as cognitivism in this paper. Consequently, this is the answer to why simple embodiment, in various degrees, is compatible with cognitivism explanations. It should be noted, however, as Chemero (2009) correctly points out, that radical embodied cognitive science is not a radicalization of embodied cognitive science. Instead, the mainstream version of embodied cognitive science, i.e., simple embodiment, could be regarded as a “watering down” (ibid., p. 30) version of the more radical scientific tradition that dates back to scholars of pragmatism. This means that the influence goes the other way around than often presented or imagined in mainstream cognitive science, and clarifies why there has been a turn to pragmatism and enactivism within more radical embodied approaches of cognition.

### Aim and Objectives

In this paper, I present and analyze several approaches of socially distributed, situated, embodied and enacted social cognition. My line of argument is that some of these approaches, although advocating toward the idea that the social mind is not merely contained within the head, fail to describe a radical embodiment view of the social mind. In the more positive parts of this paper, I suggest that this quest can be achieved by drawing upon the more radical and intersubjective accounts of embodied social cognition, which in several ways emphasize anti-representational explanations. It also shift the focus from the individual mind in social cognition to instead focus on what happens in the social interaction as such between interacting individuals in meaning-making practices, including languaging. A thesis that is emphasized is the idea that human cognition by nature is *relational*, in which the social and cultural scaffolds that human embodied beings are situated within and enculturated to, is the driving force for the emergence of our embodied social understanding and the human mind. Finally, in line with those arguments, I present some ideas and motivations for how to study and expand the field of radical embodied social cognition in the future, as well as pose the ubiquitous hazard of falling back into cognitivism in several ways.

## Social Embodiment Effects, Social Neuroscience and Embodied Simulations

### Social Embodiment Effects

In the extensive literature on embodiment effects in social psychology, the work summarized by Barsalou et al. (2003) has identified four kinds of social embodiment effects, for which there is plenty of empirical evidence. They characterize social embodiment as “states of the body, such as postures, arm movements, and facial expressions, arise during social interaction and play central roles in social information processing” (Barsalou et al., 2003, p. 43).

Firstly, perceived social stimuli do not only produce cognitive states, but also bodily states. For example, it has been reported that high school students who received good grades in an exam adopted a more erect posture than students who received poor grades (Weisfeld and Beresford, 1982). In another experiment, subjects primed with concepts commonly associated with elderly people (e.g., ‘gray,’ ‘bingo,’ ‘wrinkles’) exhibited embodiment effects such as slower movement when leaving the experimental lab, as compared to a control group primed with neutral words (Bargh et al., 1996) (it should be mentioned that this study has been criticized due to problems with replication and priming, see Doyen et al., 2012). Subjects performing a lexical decision task, using verbs referring to mouth, hand or leg motion (e.g., “chew,” “grab,” or “kick”) showed increased activation in corresponding mouth, hand, leg areas of motor cortex, although no overt action or movement was required (Pulvermüller et al., 2001). Secondly, the observation of bodily states in others often results in bodily mimicry in the observer. People often mimic behaviors, and subjects often mimic an experimenter’s actual behavior, e.g., rubbing the nose or shaking a foot (Chartrand and Bargh, 1999). Moreover, mothers tend to open their mouths after their infants have opened their own during feeding (O’Toole and Dubin, 1968, and similar effects are widely documented in the literature). Thirdly, bodily states produce affective states, which means that embodiment not only facilitates a response to social stimuli but also produces tentative stimuli. Subjects rated cartoons differently when holding a pen between their lips than when holding it between their teeth (Strack et al., 1988). The latter triggered the same musculature as smiling, which made the subjects rate the cartoons as funnier, whereas holding the pen between the lips activated the same muscles as frowning and consequently had the opposite effect. Moreover, bodily postures influence the subjects’ affective state; e.g., subjects in an upright position experienced more pride than subjects in a slumped position (Stepper and Strack, 1993). Fourthly, compatibility between bodily and cognitive states enhances performance. Several motor performance compatibility effects have been reported in experiments where subjects responded faster to ‘positive’ words (e.g., ‘love’) than ‘negative’ words (e.g., ‘hate’) when asked to pull a lever toward them (Chen and Bargh, 1999). Additionally, subjects holding warm coffee were more likely to evaluate an imaginary individual as warm and friendly than those subjects holding cold coffee (Williams and Bargh, 2008). In another study, passers-by evaluated job candidates by reviewing the resumes on either light or heavy clipboards. Participants with heavy clipboards rated the candidate as better overall and specifically as displaying more serious interest in the position. These participants also rated their own accuracy on the task higher than participants using the light clipboard (Ackerman et al., 2010).

Other research focuses explicitly on traditional conceptions in social psychology, such as attitudes, social perception, and emotions (Niedenthal et al., 2005). Niedenthal et al. (2005) suggest that social-information processing involves embodiment, with which they refer to “actual bodily states and to simulations of experience in the brain’s modality-specific systems for perception, action, and introspection” (Niedenthal et al., 2005, p. 184). They address these topics from *online* (i.e., perceiver interacts with actual social objects, e.g., mimicking a happy facial expression) and *offline* (i.e., perceiver represents social objects in their absence, e.g., understanding the concept happiness or recalling a happy experience) cognition. They argue that distinguishing between online vs. offline is helpful in systematizing the findings within social psychology, and besides, it can function as a way to conceptualize the acquisition and the use of knowledge, as well as hopefully recognizing similarities between their underlying embodied mechanisms. They provide empirical findings from three identified categories.

First, embodiment of attitudes concerns the acquisition and processing of attitudes, emphasizing that empirical studies show that bodily postures and motoric activities, such as nodding heads (in agreement) or shaking heads (in disagreement) are related with positive or negative preferences and action predispositions toward objects (Tom et al., 1991). When participants offline generated the names of famous persons (e.g., ‘Jane Fonda’ or ‘Clint Eastwood’), and then classified the celebrities according to whether they liked, disliked or were neutral about them, during the generating names phase, participants were instructed to either place their hands beneath the table and pushed upward (inclining an approach behavior) or on top of it and pushed downward (inclining an avoidance behavior). As a result, the participants directed to conduct an approach behavior named more celebrities they liked, whereas those that performed an avoidance behavior named more they disliked (Förster and Strack, 1997). Secondly, embodiment of social perception, is reported in the finding where mothers open their mouths in response to their infants’ mouth opening during feeding. One example of a reported offline effect is when researchers created descriptions of fictional characters, based on personality descriptions of significant others the participants liked in their ordinary lives. Later, in the experimental situation, while the participants read the descriptions of the fictional characters, they tended to display positive facial expressions. When the participants instead read descriptions of the fictional characters based on persons they disliked, they were inclined to show negative facial expressions (Andersen et al., 1996). Thirdly, many examples of embodiment of emotions are reported in the literature, e.g., when somebody fakes an injury and grimaces in pain, observers also grimace (Bavelas et al., 1986). Regarding offline embodiment in emotion, is was demonstrated that participants’ retrieval of pleasant or unpleasant autobiographical memories was influenced by the manipulation of facial expressions and postures. Adopting an erect posture and also smiling hastened the retrieval of pleasant autobiographical memories, compared to the speed of retrieving unpleasant memories (Riskind, 1984). In studies with Botox, temporary paralysis of the facial muscle that is responsible for producing a frown hindered processing, relative to pre-injection baseline, for angry and sad sentences, while processing for happy sentences was unaffected (Havas et al., 2010).

These examples, as well as many other similar and related studies (see Anderson et al., 2012; Glenberg, 2015 for a review of additional examples with links to various embodied metaphors in linguistics), demonstrate that there is a strong relation between so-called “embodied” and “cognitive” states in social cognition. In short, the bi-directional swapping between various components of an affection-resonance-emotion cycle changes automatically, both “online” and “offline,” without any conscious mediating knowledge structures (“content”) in attitudes, social perception and emotions (Lindblom, 2007, 2015a; Fuchs, 2018). Instead, Fuchs (2018) interprets the above social and emotional embodiment effects as an intercorporeal resonance, which favors an enacted perspective.

It has been suggested that mirror neurons function as the neuro-biological underpinning for these social embodiment effects or intercorporeal resonance and then embodied simulations may provide an embodied account of social understanding, without a grounding in internal representations, as discussed in more detail in the following section.

### Social Neuroscience and Embodied Simulation Theories

More detailed accounts of how the sensorimotor structures of the brain are involved in social cognition have been developed in several disciplines, often taking into account data from neurophysiological and neuroimaging studies. Fuchs (2018) argues that the continuous circular interaction emerges into the phenomena of emotional experience that cannot be solely located in the brain, as usually explained, but instead spans the whole body. Findings in social neuroscience provide strong evidence for a radically embodied interpretation of social understanding. Such an understanding may rely on the discovery of special kinds of visuo-motor neurons in the premotor cortex in the brain of macaques, so-called *mirror neurons*, which exemplify how perception and action might come together at the level of single neurons. Mirror neurons located in area F5 in the monkey brain become activated both when *performing* specific goal-directed hand (and mouth) movements and when *observing* or *hearing* about the same actions (e.g., Gallese et al., 1996; Rizzolatti et al., 1996). Because mirror neurons respond in both conditions, it has been argued that the mirror system functions as a kind of direct connection between ‘action’ and ‘action-perception.’ The succeeding disclosure of a mirror neuron system in the human brain (Gallese et al., 2004; Rizzolatti and Sinigaglia, 2010) demonstrates a *relational* character and reveals how the brain can map (not represent) intentional actions, implying, in turn, how deeply intertwined action, perception and cognition actually is (Gallagher, 2005; Gallese, 2017). This means that the mirroring mechanism enables the agent to grasp the meaning of the observed action by embodied (re)-activation without using internal representations. This means, even while only *observing* the actions of another individual, a neural ‘triggering’ event in fact takes place without any mediating representation in the observer, providing an ‘intuitive’ social understanding of the observed action (Lindblom, 2007). Subsequent work on the activation of the mirror neuron system has been performed in specific contexts, e.g., before and after drinking tea to investigate the understanding of intentions of others while watching their actions in different conditions (Iacoboni et al., 2005). Human subjects were exposed to three different stimuli; grasping hand actions without a context, context only, and in two different contexts (either ‘drinking – to have tea’ or ‘cleaning – after having tea’). The obtained fMRI data shows that actions embedded in contexts generated a significant increase of activity in the pre-motor mirror neuron areas of the brain, indicating that the mirror neuron system is also involved in grasping the intention of others automatically. This means, the role of the mirror neuron system seems to be more complex than mere action-recognition, otherwise a similar response should have been displayed while watching grasping actions regardless of whether the context of the observed action was present or not. Furthermore, there were different activations between the ‘drinking’ and ‘cleaning’ contexts, which imply there are certain neurons in the human inferior frontal cortex that particularly ‘grasp’ the *why* aspect of the action. Thus, the study indicates that certain kinds of mirror neurons, so called logically related mirror neurons, may constitute the foundation for more advanced forms of bodily intentionality. The description of an action and the interpretation of the reason why that particular action is performed have been considered to rely on two different mechanisms in cognitivism. The mirror neuron system, however, provides an alternative solution, given that the logically related mirror neurons automatically trigger the motor acts that are most expected to follow the observed action in the particular context (Iacoboni et al., 2005). This means, the ability to infer the forthcoming new goal is already ‘there’ in the mirror neuron system. Hence, explaining intentionality by two different mechanisms is both unnecessary and biologically implausible in regards to parsimony. In other words, the cognitive processes that are achieved by the reactivation of the same neural structures used for physically sensing, moving and acting in the environment, is also used in sense-making/meaning-making activity in social perception, social interaction and social understanding. It has been speculated that the mirror neuron system might be a basic direct mechanism necessary for imitation and grasping others’ intentions (Gallese et al., 2004; Rizzolatti, 2005; Rizzolatti and Sinigaglia, 2010; Gallese, 2017; Fuchs, 2018). As Rizzolatti (2005) and Fuchs (2018) point out, however, it is obvious that the mirror neuron system itself is unable to explain the whole complexity of speech, human language, intentionality, theory of mind, and mindreading, but actually clarifies one of the fundamental aspects of social interaction and communication, namely how the interacting partners are able to *directly share* the communicated meaning between them.

It is argued that the mirror neuron system serves as the underlying mechanism that enables the agent to understand the meaning of the observed action by so-called *emulation* or *simulation theories*, and there exist several approaches that address the social dimension (e.g., Gallese, 2004, 2005; Gallese et al., 2004; Svensson et al., 2007). Gallese’s (2004) theory of the shared manifold of intersubjectivity proposes that all kinds of interpersonal relations, such as imitation, mind-reading, theory of mind, and empathy, depend, at a basic level, on the foundation of a shared manifold space, which then is characterized by routines of embodied simulations. Gallese (2004) addresses this issue from both an evolutionary perspective as well as from current findings in cognitive neuroscience, arguing “there is now enough empirical evidence to reject a disembodied theory of the mind as biologically implausible” (p. 166). This implies that during the course of ontogeny, the mirror neuron system and the embodied simulation processes might develop further, through maturation as well as socially and culturally scaffolded interactions, to more advanced forms of social interaction and social understanding, and language (Lindblom, 2007, 2015a).

## Simple Embodiment and the Enactive Turn: Cognitivist Pitfalls in Social Embodiment Effects

The presented selected examples in the Section “Social Embodiment Effects,” and additional ones in the extensive literature on social embodiment effects, provide a positive turn to consider the role of the body in social interaction and cognition. Barsalou et al. (2003) and Niedenthal et al. (2005) offer a framework of embodied simulation to explain the underlying mechanisms for the social embodiment effects, which is based on and slightly modified from Barsalou’s (1999) Perceptual Symbol System (PSS). Pouw and Looren de Jong (2015) mention that the common strategy used in Barsalou et al.’s (2003) and Niedenthal et al.’s (2005) explanations is the mapping of the offline cognition into online cognition, triggering embodied simulation from social stimuli. The provided framework and the explanation offered is aligned with cognitivism, since it focuses on social perception, social information-processing, and social representations (although in a modal or perceptual format) rather than authentic socially situated interaction, ignoring the social affordances in dynamic social interactions in the wild. Barsalou et al. (2003) and Niedenthal et al. (2005) still continue to explain social cognition largely in terms of internal representations and the computational processes manipulating them, which adds a socially embodied icing to the traditional information-processing cake. Wilson and Golonka (2013) argue that this kind of research remains business as usual, with a couple of embodied ‘bells and whistles’, because all the hard work of generating behavior is done in the brain, it is just that this work can be biased by what the body is up to, i.e., simple embodiment.

It is argued that these strands are compatible with a ‘simple’ approach to embodiment, because studies that manipulate the subjects’ bodily cues provide a narrow scope of embodiment that lack ‘rich’ social interactions unfolding and embedded in ecological practices, being aligned with simple embodiment (Semin and Smith, 2002, 2013; Goldman and de Vignemont, 2009; Marsh et al., 2009; Durgin et al., 2012; Meier et al., 2012). Marsh et al. (2009) present a roadmap toward more radically embodied social psychology research, in which the mere importance of socio-cultural situatedness (e.g., Hutchins, 1995) and human understanding is distributed across several individuals, instead of being localized ‘in the head.’ Their approach is theoretically grounded in Gibson’s (1979) ecological psychology, where the relational meanings of the concept of affordances is central. The relationships are detected and enacted through the accurate body’s physiology and a history of interactions. Marsh et al. (2009) also stress the importance of identifying general dynamical principles that coordinate and interconnect among elements in the emergence of meaning of social behavior, stressing that the unit of analysis should shift beyond the individual level to a systems level. These suggestions have several methodological implications for the envisioned study of action and body in the environment from a more *embedded* perspective in social psychology. They offer four suggestions for how to study body-based phenomena in relation to the affordable physical and social environment. First, is an increased interest in the study of ‘doing,’ from a more functional perspective of bodily actions. Secondly, is studying how behavior unfolds in time to examine the emergence of phenomena that are the outcome of persons’ embeddedness in their environment. Thirdly, is an increased focus on joint participation in goal-directed actions, where the cooperation in joint participation in physical action is studied on both the individual and social levels. Fourthly, is studying the behavior of individuals in natural settings and investigate how humans attend to the affordances (features) in the environment and how these have an impact on behavior through the ways humans are creating and changing the environment to better fit their actions.

## The Enactive Turn: Toward Intercorporeality, Interaction Theory and Critical Neuroscience

When it comes to embodied simulations, Gallagher (2005, 2019) stresses that a radically social mind does not need any kind of embodied simulations as in the proposed versions of embodied simulation during online cognition (Gallese, 2004, 2005, 2017; Svensson et al., 2007). Instead, he argues that the understanding of the other person is *primarily* neither theoretical nor based on an internal simulation, since it is a kind of *embodied practice*. It should be noted, however, that Gallagher does not deny the cases when we use the ability of theoretical interpretations or/and simulation, since these occasions are, according to him, rather rare in proportion to the majority of our social interactions (Lindblom, 2007, 2015a). This means that embodied simulations at best explain some narrow and specialized situations of the social mind, which only *sometimes* are used in social interactions. Indeed, he advocates that in the cases when we lean more on advanced strategies, they are already shaped by primary embodied practices (Gallagher, 2005). The major problem, according to him, is the assumption, in both cognitivism and embodied simulations, that interaction and social understanding between two people is a process that takes place between two ‘Cartesian minds.’ By ‘Cartesian minds,’ Gallagher refers to the view which requires that one’s understanding usually involves a retreat to a realm of ‘theoria’ or ‘simulacra’ into a set of internal operations that becomes decoded and externalized in another modality such as speech, gesture, or action. That is, there is always some kind of higher level processing (which is using some kind of “content” and representations) being carried out in cognition (Lindblom, 2007, 2015a). Similarly, Maturana and Varela (1987, p. 196), point out that the traditional metaphor of communication is wrong, since “biologically, there is no ‘transmitted information’ in communication.” In a similar line, Shanker and King (2002) argue that the information-transmission metaphor fails to reveal the full story of social interaction, because it significantly oversimplifies and misrepresents what actually happens in social interaction. This view is aligned with Fogel (1993, p. 76) who states that “information is created in the interface between perception and action … It is that last point, the salience of the body … that is missing in many theories of meaning.”

Gallagher (2005) argues that communication is accomplished in the very action of pragmatic embodied interaction, through the expressive movement of speech, gesture, and the environmental and contextual factors of the *interaction* itself. Therefore, the idea that the understanding of another person involves an attempt to theorize an unseen belief or simulate in mind-reading is challenged. Instead, he proposes that only when our ‘second-person pragmatic interactions’ or our evaluative attempts to understand others break down do we choose to use more specialized practices of third-person explanation and prediction, i.e., embodied simulation as such is mostly carried out offline, not online, using the vocabulary used by Niedenthal et al. (2005). I emphasize that it is of major importance to be aware of the different perspectives in these situations. This means, in order to interpret and understand other people in real-time interaction, Gallagher (2005) suggests that humans seldom need to move beyond the present embodied and expressive actions at hand in order to grasp and gain an understanding of the other person. In this regard, there is not any discrete process that involves perception plus simulation, but rather a direct intersubjective perception of what the other is doing (Gallagher, 2007). He argues that phenomenologically, when one sees another person’s action or gesture, one *directly perceives* or immediately ‘sees’ the meaning in the action/gesture, without the need to simulate it. He presents brain-imaging studies, in which subjects were asked to simulate their own movements (first-person perspective) or another person’s (third-person perspective) movement. The result shows that there is no additional brain activity in favor of an extra level or effort as a kind of simulation, meaning there is no evidence for viewing simulation as an ‘extra’ step (cf. e.g., Gallese, 2004) over and above the perception. Indeed, Gallagher’s point is “that there is no evidence that perception and simulation are two separate systems. In other words, the neurological underpinnings of what could count as embodied simulations are part and parcel of the (re-)activations that correspond to the original perception from an embodied pragmatic perspective (Gallagher, 2005). This poses another problem, however, namely where to draw the line between perception and other (cognitive) processes. Subsequently, the need of an internal model is questioned, and as Gallagher (2005) explains, “[t]he required model *is* the action of the other, and it is already being perceived. Why would one need to ‘read off’ the meaning of an action on an internal ‘as if’ model, indirectly, when one is observing that very action performed by the other?” (ibid., p. 224). Gallagher (2007) mentions that proponents of embodied simulations stress that simulation involves the instrumental use of a first-person model to form third-person “as if” or “pretend” mental states, but he argues that this is not a possible explanation. He explains that we cannot control these re-active sensorimotor processes at a personal level, and for that reason we cannot use them as a model. Thus, there is no homunculus present. Another proposed idea that the brain itself, at a subpersonal level, is using these reactivations as a model (cf. Damasio, 1995), which does not make sense either according to Gallagher (2007). Thus, his major point is that “the neural systems neither activate themselves nor take the initiative, but are *activated by the other person’s action.”* Thus, “the other person *has an effect on us.* The other elicits this activation… It is not us (or our brain) doing it, but the other who does it to us” (ibid., p. 8–9). Gallese’ s (2014) reply to Gallagher’s criticism of embodied simulations is that he interprets mirror neuron mechanisms and embodied simulations as instantiations of neural *reuse*, i.e., the dual firing/activation pattern of a certain group of mirror neurons in a certain situation, in which they either are executing an action or observing an execution by others. Gallese (2014) claims that according to this view, mental representations are entirely not required. Gallese (2014) suggests that with a foundation in the mirror neuron systems and by means of neural reuse, embodied simulations is an elemental way to comprise the “representation of the motor goals of others’ actions by reusing one’s own bodily formatted motor representations, as well as of others’ emotions and sensations by reusing one’s own visceromotor and sensorimotor representations” (Gallese, 2014, p. 7). According to Gallese (2014, 2017), embodied simulations therefore could offer a unified explanatory framework for social understanding, mindreading, theory of mind and cognition. However, Gallagher (2015, 2019) seems not to agree, and there is an ongoing discussion between these two scholars whether there is any room for representations (Gallese, 2017 denotes them B-formatted representations) or not in social interactions and social understanding. In a nutshell, from a radical embodiment perspective, it is desirable to reduce, or even ignore, the role of mental or internal representations altogether (Gallagher, 2005, 2007, 2015, 2019; Hutto and Myin, 2017), a stance that has been criticized by others (e.g., Gallese, 2004, 2005, 2014, 2017).

It should be pointed out that the term *mirror neuron systems* could be leading us astray, because the term implies almost extraordinary abilities of single neurons in the form of achieving social perception by themselves, and these neurons may not be able to react to aspects of more complex situations of social perception and interaction (Fuchs, 2018). These concepts also ascribe a representational view of the mirror neuron system and embodied simulations, *via* some kind of internal imaging that is re-produced or “mirrored/simulated” onto the other. Therefore, Fuchs (2018) prefers to use the term social resonance system, because these “*neurons cannot mirror* [or simulate] *anything”* (p. 187). Consequently there is no representational image or simulation to be found. He argues that a mirror only reflects rays of light, and in order to perceive this light as a mirror image you need to be an embodied and conscious being, and there is no need to simulate anything. Indeed, the mutual linking between action and perception offers an ‘intuitive’ understanding of the observed action, i.e., what it means to do it, how it “feels” in the body and what the action really is about and for what purpose for the agent. Fuchs (2018) emphasizes that tentative interpretations of the social resonance system are that it contributes to perceive movements of conspecifics in terms of *goal-oriented actions*, intermodal connections that support action readiness, providing the basis for imitational learning. Thus, the social resonance system provides an *operative intentionality* of our body as a means to understand the intentional movements of the other agent, since our body by itself —without any representational content— “resonates” these into our own actions. This way of reasoning is aligned with Mearleau-Ponty’s concept of *intercorporeality* (Fuchs, 2018). In other words, *intercorporeality* allows us to continuously perceive others as our own kind since our body is subliminally attuned to the others’ gestures, facial expressions, emotions, and the intentions of their movements and actions through “interbodily exchange,” without primarily being based on representational concepts as mindreading and theory of mind abilities as proposed by cognitivism (Fuchs, 2018).

At this point it should be noted that for the purposes of this paper, the jury is still out when questioning whether or to what extent the role of embodied simulations matters in radically embodied explanations of social understanding. I would like to emphasize, however, that embodied simulations theories offer a much more radically embodied explanation than representational conceptions of “mind-reading” and “theory of mind,” because they stress the directly experienced embodied perception of intersubjectivity and social resonance of other human beings, without the need to create an internal symbolic model or mirror of the other person. I will not continue to discuss this issue here in more detail.

Instead, I shift the focus of my arguments on tentative ways of opposing body snatching (Gallagher, 2015) by stressing the claim that the brain, from a radical embodiment perspective, should be considered as a vehicle for action and its should be better to study its functions at the level of the whole brain-body-environment system (Kiverstein and Miller, 2015; Fuchs, 2018). A tentative approach to bridge the troubled water of radical embodiment in cognitive neuroscience and phenomenological experience is the raised quest for a pragmatic and radical embodied neuroscience (Engel, 2010; Kiverstein and Miller, 2015; Slaby and Gallagher, 2015; Di Paolo et al., 2017). Engel (2010) points out that there is plenty of evidence that supports the pragmatic and enactive view by findings on the important role of sensorimotor interactions and explorative activity for the neural development and brain plasticity. He mentions that it has been acknowledged for quite a long time that the nervous system’s developmental processes are highly dependent on various kinds of activity. Engel (2010) envisions a conceptual shift toward a “pragmatic neuroscience,” which in due course will result in different style of experimentation, and setting the scene for new “laboratory habits” (Engel, 2010, p. 237). An increasing number of researchers have begun to use more natural and contextual stimuli, and using more active subjects in the lab studies, since “world-making” rather than “world-mirroring” lies at the heart of enacted cognition (Di Paolo et al., 2017; Fuchs, 2018). Kiverstein and Miller (2015) outline and explain why a radical embodied cognitive neuroscience is considered necessary. They address the concept of the “embodied brain,” arguing that neuroscience should turn more to Gibson’s (1979) ecological approach to get a better grasp of the cognitive functions that the brain performs. They stress that there is a need for a shift from focusing on localizing different cognitive functions to specific brain structures, which they find problematic, to describing and studying cognitive functions at network levels of the whole interactive brain-body-system. They envision that the main contribution of applying such a system view, regulated through the organism’s interaction with the environment, affords several possibilities for actions. Thus, their major claim is that cognitive functions in the brain is *context-sensitive*. For example, Lifshitz et al. (2017) mention that neuroscientific findings demonstrate that bodily posture, e.g., being upright versus lying down, profoundly alters baseline brain activity when measured by magnetoencephalography (MEG). Kiverstein and Miller (2015) also address the intimate interrelatedness of cognitive and emotional processes in the brain, stressing that emotions are dynamical and encompass the whole body of an organism that is engaged with its environment, in which emotions influence the regulation between the organism and the environment. Similar arguments have been proposed by Stapleton (2013) who advocates that human beings are “properly embodied,” which means that sensorimotor interaction with the environment is not enough, the internal bodily system also matters to cognition. She suggests that the relationship between cognition and affect is more complex and important than previously understood, implying a more organismic and enactive paradigm of embodied cognition. In such a properly embodied cognitive science, the affective system is integrated in cognition in itself (Stapleton, 2013).

Additional enactivism accounts for what role the brain has in social cognition, if not being representational, and is known as the *interactive brain hypothesis* (IBH) (De Jaegher et al., 2016).

The starting point for IBH is that social cognition also needs causal relations between the brain and the *social environment*, and should include how several kinds of cognizers experience and grasp the world as meaningful in various situations, but also to take an interdisciplinary approach that spans developmental and evolutionary perspectives. IBH also offers a guide how to study social interaction, e.g., identifying what kinds of social events and social relations, kinds of brain activities, and certain instances of social cognition. They conclude that because there is a development of methods and techniques for examining activities in the brain during more free interactions than before, it is necessary to hypothesize about these questions, when the upcoming and envisioned brain studies may include joint actions and emergent collective patterns distributed over multiple brains/bodies/persons in several kinds of coordination (De Jaegher et al., 2016). Thus, the take-home messages under the banner of radically embodied and enactive neuroscience are twofold; first, do not consider the individual biological system (human or animal) that is studied experimentally for the fully embodied person. Second, try to find ways that encompasses the practices of socially situated and embedded humans in society (i.e., striving for ecological validity), otherwise do not claim that current social neuroscience approaches are able to study the human social mind in its full scope (Slaby and Gallagher, 2015).

Recently, Fuchs (2018) offers a tentative embodied and enactive perspective on the role of the brain in cognition. He presents a view of the human brain that goes beyond neurobiological reduction, in which the brain does not produce the mind. Fuchs portrays a convincing and detailed approach of the brain, emphasizing that the brain is an *organ of mediation and integration*, rather than of information-processing of mental representations. The human brain is “alleged to bring fourth … conscious human persons who exist to communicate with each other. It is indeed the case that and neuroscience cannot escape its inherent dependence on subjectivity, intersubjectivity, and the lifeworld … [it is] the familiar world of everyday experience in which we coexist with others remains our primarily and actual reality” (Fuchs, 2018, p. xix). If we take this stance, the common view of the brain as an invisible creator of mind or the place where the subject is located needs to be abandoned in favor of the function of the organ of the lived body that mediates our relationships with the surrounding, other people, and last but not least to ourselves. Fuchs (2018) reformulates the dichotomy of the mind (mental)-body (physical) problem into a *dual character of life* that is manifested in the entire body as a living organism. In Fuchs’s enacted theory of *dual aspectivity*, the living being itself, i.e., the whole human being not the brain only, is the primary entity, in which the manifestations of life are considered. On the one hand, of the integrated subjective and intersubjective acts of the *lived body*, and on the other hand, of the physiological processes of the *living body*. The embodiment of a human being’s life, through its dual (not dualistic) aspects of *lived* and *living body*, is the dialectic mediating entity through which the aspects of subjectivity and nature are interrelated and complementary, but do not completely overlap. The life acts (e.g., speaking, suffering, eating, playing) of the subjective lived body could be experienced from first (inner) as well as perceived from second (outer) perspectives of the person. Fuchs denotes the latter perspective as a “we-perspective,” in which we perceive each other as living human beings and not objects. This means that the embodied (emotional, cognitive, or volitional) acts of the living body are not assigned to the sole “mental” sphere, because they are always embodied physical events. The physiological processes of the *living* or *objective body* could only be perceived from the third-person perspective that corresponds to the whole living organism’s interactions with the material and social world. Thus, the complementary nature of the living and lived body could be considered as two sides of the same coin, where only one of them is visible at the current moment. Thus, all experiences are a form of living, where the whole human is an ontological and fundamental being-in-the-world (Fuchs, 2018). This means that the brain does not operate in isolation, because it is an organ of interrelations that spans the human person as the unit of a living organism, and could only be explained from that perspective.

This line of argument is well-aligned with radical embodiment and the enactive approach, in which social cognition is characterized by, and very often constituted by socially embodied interaction, and the dual aspect of the lived body (e.g., Sinha and Jensen de Lopez, 2000; Gibbs, 2001, Gibbs, 2006; Lindblom, 2007, 2012, 2015a,b; Lindblom and Ziemke, 2007, 2008; De Jaegher et al., 2010; Fuchs, 2018). This turn to the social and relational sphere is the topic for the next section.

## The Intersubjective Turn: Participatory Sense-Making and Linguistic Bodies

The appeal for a social dimension of radical embodiment has been pointed out by several researchers (Maturana and Varela, 1987; Fogel, 1993; Sinha and Jensen de Lopez, 2000; Gibbs, 2001; Shanker and King, 2002; Lindblom and Ziemke, 2003, 2007, 2008; Johnson, 2007; De Jaegher et al., 2010; Lindblom, 2012, 2015a,b; Cuffari et al., 2015; Slaby and Gallagher, 2015; Di Paolo et al., 2018). Although the social sphere of radical embodiment has not been mentioned explicitly so far in this paper, I will now explain *how* the social mind at an individual level is realized, and ways of describing and incorporating the individual social mind at the social level.

It should be noted, however, that there are two major problems with cognitivism that I want to address before taking the more radical turn on the social mind (Lindblom and Ziemke, 2008; Lindblom,2015a). First, as already touched upon earlier, cognitivism considers human communication and social cognition as mostly exclusively private mental states in individual minds that ignores the dynamic, interactive, and subjective nature of intentional actions. This position is referred to as “methodological individualism: the assumption that social cognition depends on capabilities or mechanisms within an isolated individual, or on processes that take place inside an individual brain” (Froese and Gallagher, 2012, p. 437). However from a more embodied and enactive perspective, it is generally stressed that social interaction cannot be reduced to so-called ‘social information transfer’ (see sub-section “Social Embodiment Effects”). My main point here is that information is not an identified and discrete entity which can be sent, through signals, from one person across time and space to another person. Taking a more pragmatic and enactive turn, several researchers focus on the emergence of *meaning-making* or *sense-making* in the dyads and triads between humans, in which dynamically emerging, creative co-regulated socially embodied interactions serve as the basis for social understanding and social cognition. Secondly, Gibbs (2001) provides a tentative explanation for the methodological individualism within cognitivism from work in cultural anthropology. He emphasizes that the main focus on an individual’s intentions in social interaction and cognition by most scholars rather reflects a Western white middle-class bias about the nature of selfhood than a universal phenomenon, because the underlying assumption of the individual mind is a view not shared across different cultures. Hence, it might be argued that individual intentionality is one of the ‘holy cows’ of Western thought. Thus, focusing too much on the assumption of methodological individualism, overemphasizes the individual’s psychological state at the expense of the social and cultural context in which the actions unfold (Gibbs, 2001).

However, I will argue that many of the radical embodiment and enactivism approaches (Di Paolo et al., 2017; Hutto and Myin, 2017), as currently formulated, to some extent suffer from the same limitations as Piaget (1952, 1954) developmental theory, not paying sufficient attention to the role and relevance of culture and society in social cognition (Lindblom,2015a). Piaget’s main focus was on the individual child’s construction of its reality, where he identifies three kinds of knowledge, each of them resulting from the child’s interactions with the environment, namely physical, logical-mathematical, and social knowledge. In Piagetian terms, the child first develops as an individual being, and later on into a social being (Lindblom, 2007, 2015a). This is contrary to the Vygotskian approach, which views the child’s individual development as the outcome of the social interaction of the human species and the child’s interactions with other people in their particular culture. In his *general law of cultural development*, it is stated that *“*every function in the child’s development appears twice: first, on the social level, and later, on the individual level” (Vygotsky, 1978, p. 56). In this section of the paper I will strongly emphasize the importance of the social context, which I refer to as *relational*, which has strong roots in the work of Dewey (1896,1925/1981), Mead (1934), and Vygotsky (1977, 1978, 1979). I do not have enough space here to elaborate in more detail on how these above scholars in the late 19th century and the beginning of the 20th century emphasize that the human cognition and social understanding emerges and is enacted through social interactions, although they put varying emphases on the role of the embodiment (but see Lindblom and Ziemke, 2003, 2006; Lindblom, 2012; Lindblom,2015a). To provide but one example, I present Vygotsky’s (1978) account for the development of pointing in the child to illustrate the *relational* aspect of social interaction. He claimed that initially it is only a simple and incomplete grasping movement directed toward a desired object, and nothing more. When the caretaker comes to help the child, the meaning of the gesture situation itself changes, by obtaining another meaning, as the child’s failed reaching attempt provokes a reaction, not from the desired object, but from the other person. The individual movement ‘in itself’ in its social context becomes a gesture ‘for-others.’ The caretaker interprets the child’s reaching movement as a kind of pointing gesture, resulting in a socially meaningful communicative act, whereas the child their self at the moment is not actually aware of the communication ability. However, after a while the child becomes aware of the communicative function of their movements, and then begins addressing its gestures toward other people, rather than the object of interest that was their primary focus initially. Thus, “*the grasping movement changes to the act of pointing”* (Vygotsky, 1978, p. 56). This means, the intention of pointing does not reside within the child’s individual mind, it emerges as an outcome of their on-going social interactions. Accordingly, by treating the child as an intentional being, caregivers ‘bootstrap’ and scaffold them into a socio-cultural environment, which partly rests on the ‘illusion of intentionality.’ The grasping example illustrates the role and relevance of social interactions and shared practice of whole embodied persons, especially during childhood, in the form of *embodied intersubjectivity* and *communicative intercorporeality* as a prerequisite for the emergence of the full-blown human mind. It should be mentioned that the newborn infant’s brain possesses a unique potential, which requires not only interactions between brain body and environment, but also with other *human beings*, to realize the development of the embodied and enacted social mind. Fuchs (2018) addresses that these interactions form traces at a neural level, but not in the form of stored and localizable “representations,” “memories” or “intentions” of the actions, but rather as “*dispositions to perceive, feel, and behave in certain ways”* (p. 181). These dispositions consist of a distributed network of neural connections, which resonates with the current situation at hand as well as other human beings. Today, many scholars follow in Vygotsky’s and his followers footsteps by emphasizing that the human mind and advanced social understanding transcends the biological level, and that the shared social and cultural spheres of other human beings, are only acquired by active participations in these ecological practices. It is argued and shown that enculturation is of outmost importance for humans compared to any other species. Fuchs claims that all so-called higher cognitive functions “presuppose the *human being’s enactment of life in a shared social world*” (p. xx, original emphases). These interactive and intersubjective experiences form the foundation for acquiring and internalizing the dispositions of the interactional patterns, cultural symbol systems, language, and social understanding in the child’s society, and has a much stronger impact on social, emotional and cognitive abilities than was understood in previous research on human development. Fuchs (2018) points out that embodiment is the basis for corporeal resonance and intersubjectivity with other human beings, and the explanations of “mind-reading” and “theory of mind” concepts used in contemporary social cognitive psychology are misleading in several ways. He advocates a kind of cultural biology which is well aligned with Donald’s (1991), Tomasello’s (1999), and Rogoff’s (2003) thesis that humans are “biologically cultural.” Thus, ‘culture’ reinforces ‘biology’ as much as ‘biology’ reinforces ‘culture,’ which means that the divide between ‘culture’ and ‘biology’ is an artificial abstraction in human ontogeny and phylogeny. It should be pointed out, however, that the above scholars, to various extents, are using a cognitivism stance, and my major issue is to raise awareness to the important idea of putting enculturation as the major driving force for human development. Hence, Fuchs’s claim that the brain is a *relational* organ and thereby enabling embodied intersubjectivity, social and cultural scaffolding that are the hallmarks of human enculturation, complements the above idea.

Lindblom (2015a, b), among others, has presented several examples of frame-by-frame analyzed images from different episodes of spontaneous social interaction captured *in situ*, analyzed from a more radical social embodiment perspective. One example is from a horse ranch that maintains and preserves Spanish mustang horses (Lindblom,2015a), where a joint action was illustrated and analyzed. The other example is from an archeological excavation of an old burial ground where meaning-making as a socially distributed joint activity was used an illustrative example (Lindblom,2015b). This kind of work illustrates how meaning-making activity emerges from bodily mediated and socially distributed actions. Accordingly, meaning and emotional significance is co-constituted in the interaction – not in the private boundaries of one or the other person’s head (or brain) (Gallagher, 2016).

Although the above work by Lindblom (2015a, b) is a promising step in the right direction, it does not fully take the enactive interaction process as its point of departure. De Jaegher and Di Paolo (2007) offer a basis for a more detailed enactive interpretation of social cognition by extending the enactive concept of sense-making into the social sphere. Their starting point is the interactive process between individuals in social situations, following in the footsteps of Varela’s et al.’s (1991) framework. Five core ideas, which are mutually supporting, defining the enactive paradigm are used, which are the concepts of autonomy, sense-making, embodiment, emergence, and experience (De Jaegher and Di Paolo, 2007; Di Paolo et al., 2010). Their novel notion in the social sphere is referred to as *participatory sense-making*, in which the responsibility of social understanding is moved beyond a single individual (De Jaegher and Di Paolo, 2007). Thus, the unit of analysis is expanded to the social interaction itself. They aim to figure out what the interaction process does for social cognition, by properly considering the situatedness and embodiment of the individual as well as not being ‘methodologically individualistic.’ The main topic for their participatory sense-making approach is to clarify *why* and *how* people interact, reducing the gap between the cognitive science and social science perspectives, characterizing how the individual and social levels are interrelated. For example, they mention that Gallagher’s ‘embodied practice of mind’ Gallagher (2005) does not yet provide the richness of the social interaction process and its role in developing social understanding. In doing so, De Jaegher and Di Paolo (2007) suggest that *correlation* and *coordination* is the main mechanisms of social interaction, where interactional coordination, functional coordination, and interaction rhythm (timing), and rhythmic capacity are deliberations of these mechanisms. Consequently, they define social interaction as follows: “Social interaction is the regulated coupling between at least two autonomous agents, where the regulation is aimed at aspects of the coupling itself so that it constitutes an emergent autonomous organization in the domain of relational dynamics, without destroying the autonomy of the agents involved (though the latter’s scope can be augmented or reduced)” in the process (De Jaegher and Di Paolo, 2007, p. 493).

They emphasize that the generation of social meaning is dependent on the individuals’ sense-making process itself, in which the process of coordination between actions involved in participatory sense-making contributes to people’s understanding of each other. In this way, social understanding is enacted – brought about within the interaction, supported and constrained by the elements and dynamics of interaction between the cognitive agent and the environment. As a result of the great importance of autonomy within the enactive approach, the social agent is an active participant within this unfolding process, and not a mere passive observer (De Jaegher and Di Paolo, 2007). They describe that throughout the engagement in the joint process of sense-making between at least two individuals, meaning is created and transformed *via* emergent patterns of coordination and breakdowns, which proceed to develop collective properties *via* stabilized patterns of joint activity. When the outcome from these patterns is mutually constructed, new meanings are then created in the interaction. De Jaegher and Di Paolo (2007) suggest that their dynamical and enactive view of participatory sense-making provide a novel theoretical two-way link between the individual and social perspectives, in which they envision a developmental route.

However, some problems with De Jaegher’s and Di Paolo’s (2007) participatory sense-making framework have been identified by Kyselo (2014). The first addressed issue regards the so-called body-social problem. Kyselo (2014) adopts an enactive approach to this problem, in which the problem is referred to as the quest of how bodily individual autonomy and higher, socially enacted forms of autonomy, are interrelated. Generally, Kyselo (2014) argues that participatory sense-making for social cognition is, to some extent, ambiguously formulated and explained concerning the role of social interactions for the individuation of identity. Her first concern is that the expanded unit of analysis of social interaction is a group identity, in which the whole autonomous system is more than the individual. Her second concern is that participatory sense-making also stresses the role of social interactions for the individual, by widening individual cognitive capacities through scaffolding (Kyselo, 2014). She argues that De Jaegher’s and Di Paolo’s (2007) definition of the body does not consider it as social. The identity of the individual is then defined not in social terms, but only in bodily terms. However, this is ironic, Kyselo (2014) argues, since in their very attempt to keep the individual from dissolving in participatory sense-making, they risk to reduce the role of the social. Kyselo (2014) then suggests that in order to overcome this dilemma, one has to admit that individuation of human identity is not fully determined in terms of bodies in isolation but requires that the body engages in *socially mediated* interactions. Hence, this view allows to combine both claims, stressing that individuals are *embodied interactors*. To conclude, taking an enactive approach where sense-making and autonomy implies each other, resulting in a view on human cognitive identity that is not only embodied, but primarily socially constituted (Kyselo, 2014).

The idea of participatory sense-making is extended and deepened in the enactive conception of *language* in form of a kind of adaptive, dynamical, and dialectical phenomena (Cuffari et al., 2015; Di Paolo et al., 2018). They propose that to fully encompass the phenomena of language, it must be approached in the situatedness of concrete enactments of certain kinds of participatory sense-making. They offer a detailed and comprehensive dialectical model of *languaging* that involves several steps and forms of social agency that both involves regulation of self and social interaction that encompasses the fundamental tensions that are essential in these dialogical organizations. This results in a new form of embodied agency that is denoted *linguistic bodies*. These linguistic bodies are both individual and social by nature, because they are transformed by and through the participatory use of language. This results in new forms of social autonomy, and this allows humans the transformative experience to fully participate in linguistic communities (Cuffari et al., 2015; Di Paolo et al., 2018). To summarize, the dialectical model of linguistic bodies provides a novel and much needed explanation of the role and relevance of a relational view and a socially enacted practice of language and its development from an autopoetic perspective that takes a holistic perspective that encompasses both the embodied agency and its linguistic community in society. This means that there is no inferential leap separating the embodied agency from a description of its form.

## Concluding Discussion

I opened this paper by referring to the intense and ongoing questioning concerning the role and relevance of the body in social interaction and cognition within cognitive science and related disciplines, addressing that currently there is no single, simple answer to this question. Indeed, social radical embodiment is still an emerging framework that must be coherently developed and extended, both theoretically and methodologically, subsequently resulting in richer and deeper explanations and illustrations of socially embodied and enacted actions that are situated, enacted, embedded, and carried out in practice. An issue that has not been mentioned so far is the significant role of tools and artifacts as coordinators and mediators for socially, embodied, enacted practices. In favor of this argument, there is neurological evidence for the inclusion of external tools into the body schema, spread across the entire nervous system and its couplings with the environment, rather than solely in regions of the brain (e.g., Maravita and Iriki, 2004; Cardinali et al., 2009). Although tool use is an issue beyond the topic of this paper, I wish to mention that one of the successors of Vygotsky’s (1978) work, Activity Theory, provides a broad conceptual framework for understanding and describing the structure, development, and context of human activity, focusing on the individual, artifacts, and other humans in everyday activity, as well as their interrelatedness (Leontiev, 1978, but see also the work on material engagements by Malafouris, 2013).

As a concluding remark, I would like to offer a tentative explanation to the paradox why so many accounts of mindreading concepts in folk psychological terms are present in our (Western) linguistic community, although many proponents of radical embodiment do their best to provide non-representational explanations of our social understanding of others. We as embodied agents bring fourth our own linguistic practice and habits. Therefore, it might be counterproductive to reject the folk psychological explanations of the human mind in terms of mindreading capacities, because we have enacted them by ourselves at a societal level. Johnson (2007,2018) provides a promising answer to this paradox, which is in line with Fuchs’s enacted theory of dual aspectivity (Fuchs, 2018). Johnson (2007) explains that a crucial underlying reason is that our lived experience emphasizes the dualistic view of mind and body. He argues “that our bodies hide themselves from us in their very acts of making meaning and experience possible. The way we experience things appears to have a dualistic character” (p. 2). It is therefore rather ironic that our body does impressive work for the most part “behind the scenes,” so that we as human beings can focus on the objects of our interest. This way of working results in a sense of intentionality that appears to be directed “out there” in the world (Johnson, 2007). Thus, our experience of the whole embodied organism is misinterpreted and instantiated in folk psychological terms of “beliefs,” “intentions” and so on, an enculturation process that has been manifested in our Western intellectual and cultural heritage.

Only the future will tell us whether the field of radically embodied cognitive science will expand to further directions. I would like to end this paper by seriously looking back. As pointed out by Di Paolo et al. (2017, paraphrasing Bruner, 1990), in the early inception of cognitive science in the mid 1950s, the focus soon shifted from discovering the meaning-making processes that human beings create out of their encounters with the material and social world to information processing, ending up in cognitivism. This path then lost its original target of cognitive science, since, the nuances of the phenomenological meaning and sense-making process of human beings could not be reduced to bits of information (Bruner, 1990; Johnson, 2007,2018).

## Author’s Note

Some minor content from this manuscript was published in-part for my Ph.D. thesis (Lindblom, 2007). This manuscript is an elaboration of the questions that I originally formulated and raised in the thesis, and here I present the further development of these questions.

## Author Contributions

The author confirms being the sole contributor of this work and has approved it for publication.

## Conflict of Interest

The author declares that the research was conducted in the absence of any commercial or financial relationships that could be construed as a potential conflict of interest.
